# Towards a comprehensive *Plasmodium falciparum* merozoite cell surface and secreted recombinant protein library

**DOI:** 10.1186/1475-2875-13-93

**Published:** 2014-03-12

**Authors:** Zenon A Zenonos, Julian C Rayner, Gavin J Wright

**Affiliations:** 1 Cell Surface Signalling Laboratory, Wellcome Trust Sanger Institute, Cambridge CB10 1SA, UK; 2 Malaria Programme, Wellcome Trust Sanger Institute, Cambridge CB10 1SA, UK

**Keywords:** *Plasmodium falciparum*, Merozoite, Recombinant proteins, Mammalian expression system, Protein purification platform

## Abstract

**Background:**

*Plasmodium falciparum* is the aetiological agent for malaria, a deadly infectious disease for which no vaccine has yet been licensed. The proteins displayed on the merozoite cell surface have long been considered attractive vaccine targets because of their direct exposure to host antibodies; however, progress in understanding the functional role of these targets has been hindered by technical challenges associated with expressing these proteins in a functionally active recombinant form. To address this, a method that enables the systematic expression of functional extracellular *Plasmodium* proteins was previously developed, and used to create a library of 42 merozoite proteins.

**Methods:**

To compile a more comprehensive library of recombinant proteins representing the repertoire of *P. falciparum* merozoite extracellular proteins for systematic vaccine and functional studies, genome-wide expression profiling was used to identify additional candidates. Candidate proteins were recombinantly produced and their integrity and expression levels were tested by Western blotting and ELISA.

**Results:**

Twenty-five additional genes that were upregulated during late schizogony, and predicted to encode secreted and cell surface proteins, were identified and expressed as soluble recombinant proteins. A band consistent with the entire ectodomain was observed by immunoblotting for the majority of the proteins and their expression levels were quantified. By using sera from malaria-exposed immune adults, the immunoreactivity of 20 recombinant proteins was assessed, and most of the merozoite ligands were found to carry heat-labile epitopes. To facilitate systematic comparative studies across the entire library, multiple *Plasmodium* proteins were simultaneously purified using a custom-made platform.

**Conclusions:**

A library of recombinant *P. falciparum* secreted and cell surface proteins was expanded by 20 additional proteins, which were shown to express at usable levels and contain conformational epitopes. This resource of extracellular *P. falciparum* merozoite proteins, which now contains 62 full-length ectodomains, will be a valuable tool in elucidating the function of these proteins during the blood stages of infection, and facilitate the comparative assessment of blood stage vaccine candidates.

## Background

*Plasmodium falciparum* is the aetiological agent of the most deadly form of malaria, an infectious tropical disease that accounts for up to one million deaths annually [1,2]*.* The vast majority of malaria fatalities (85-90%) occur in sub-Saharan Africa, primarily in pregnant women and children under the age of five [2,3]. While anti-malarial drugs exist, the emergence of drug-resistant parasite strains remains a global health concern and no vaccine has been licensed to date.

The asexual blood stages of malaria are initiated when a form of the parasite, called a merozoite, invades, replicates and synchronously ruptures host erythrocytes [4] releasing up to 32 progeny merozoites that can invade new erythrocytes. This cyclical phase causes the recurrent fevers and chills that are characteristic of malaria infection [5]. Merozoites are ovoid cells containing apically located secretory organelles that release proteins which are required for the invasion of new erythrocytes [6,7]. While erythrocyte invasion is a rapid process, the brief extracellular exposure of merozoites outside of their intra-erythrocytic niche places them in direct contact with host antibodies, which contribute to naturally acquired immunity to malaria [8,9]; therefore, merozoite cell surface and secreted proteins have long been considered attractive targets for rational vaccine development.

The publication of the *P. falciparum* genome project in 2002 [10] identified the full complement of parasite proteins but progress in understanding the function of these proteins, including those displayed on the merozoite cell surface, has been hindered by the technical difficulties in expressing *Plasmodium* proteins in a functionally active form [11]. Although the reasons why *Plasmodium* proteins are difficult to express in heterologous expression systems are not clear, several protein characteristics, such as high molecular mass (>60 kDa), presence of export motifs, and atypical signal peptide sequences negatively impact recombinant expression [12]. In addition, the remarkably high (~80%) A + T content of parasite genes can result in long stretches of repetitive amino acids [13], and codons that are not frequently used by organisms popular for heterologous protein expression. Extracellular vaccine candidates, in particular, present an additional challenge because they often require structurally critical disulfide bonds for correct folding and contain transmembrane domains that make them difficult to solubilize in detergents that retain their native conformation [14-16].

Despite these challenges, recombinant expression of *Plasmodium* proteins has been attempted in a number of expression systems [12,17] ranging from bacteria [18], yeast [14,19], *Dictyostelium discoideum*[20], plants and algae [21,22] to mammalian cells [23,24] and cell-free systems [13]. Among them, *Escherichia coli* is the most popular [17], but the systematic expression of functional *P. falciparum* proteins remains difficult, with success rates as low as just 6% [25], and often requires subsequent laborious and complex refolding procedures with uncertain outcomes [26]. Consequently, the functional characterization of extracellular parasite proteins has typically been restricted to smaller subfragments that can be expressed rather than the full-length protein or entire ectodomain, which is more likely to be representative of the native protein.

The development of a standardized method to express large panels of *P. falciparum* cell surface and secreted proteins in their native conformation would enable comprehensive protein libraries to be systematically screened in parallel so that direct comparisons between antigens can be made in functional assays such as vaccine screening and immuno-epidemiology studies. To achieve this, Crosnier and colleagues recently developed a method of expressing the entire ectodomains of functional recombinant *Plasmodium* proteins and used it to compile a large library of 42 proteins [27]. Working towards a comprehensive library of cell surface and secreted *P. falciparum* merozoite proteins, this manuscript describes the identification and characterization of an additional 20 proteins.

## Methods

### Recombinant protein design and expression

Proteins were expressed essentially as described previously [27]. Briefly, full-length secreted molecules and the entire ectodomains of membrane-embedded proteins were identified using transmembrane [28], GPI-anchor [29], and signal peptide [30] prediction software. To prevent the inappropriate addition of glycans, which are absent from *Plasmodium* proteins [31], all potential N-linked glycosylation sites (N-X-S/T, where X is not proline) were systematically mutated by substituting alanine for serine/threonine at these sites. Gene constructs were made by gene synthesis (GeneartAG) using sequences that were codon-optimized for expression in human cells. Protein coding sequences were flanked with unique NotI and AscI restriction sites and subcloned into a derivative of the pTT3 expression plasmid between a 5’ mouse variable κ light chain signal peptide [32], and a 3’ tag consisting of the rat Cd4 domains 3 and 4 followed by an enzymatic biotinylation sequence and a hexahistidine tag [33]. Proteins were expressed as soluble monobiotinylated proteins by transient cotransfection of HEK293E cells with the BirA biotin ligase [34] and harvested six days post transfection [35]. All expression plasmids are openly available from Addgene [36].

### Parallel protein purification

His-tagged recombinant merozoite proteins were purified from spent tissue culture supernatants by using a custom-built, piston-driven, sample-loading apparatus as described previously [33]. Briefly, a 96-well His MultiTrap HP filter plate (GE Healthcare) was pre-equilibrated with binding buffer (20 mM sodium phosphate, 40 mM imidazole, 0.5 M NaCl, pH 7.4) at a flow rate of 1 mL/min. The harvested tissue culture supernatants (~200 mL for each protein) were supplemented with imidazole (10 mM) before loading each well at 1 mL/min. The plate was washed with 1.6 mL of binding buffer and proteins eluted with 0.2 mL of elution buffer (20 mM sodium phosphate, 0.4 M imidazole, 0.5 M NaCl, pH 7.4).

### Enzyme-linked immunosorbent assay (ELISA)

ELISAs were performed as previously described [37]. Briefly, purified recombinant biotinylated proteins were serially diluted in PBS-T (PBS, 0.1% Tween-20) with 2% BSA, and captured on streptavidin-coated, 96-well plates (NUNC) for one hour before washing and incubating with the anti-Cd4 monoclonal antibody OX68 (1 μg/mL in PBS-T, 2% BSA) for another hour. Plates were washed and incubated with an anti-mouse IgG (Sigma) secondary antibody conjugated to alkaline phosphatase for one hour before further washes and incubation with p-nitrophenyl phosphate (Substrate 104; Sigma) at 1 mg/mL. Absorbance was measured at 405 nm on a PHERAstar plus (BMG Labtech). Concentrations were calculated by comparison to known standards.

### Western blotting

Each purified recombinant protein was resolved by SDS-PAGE under reducing conditions before blotting onto Hybond-P PVDF membrane (GE Healthcare) for one hour at 30 V. Membranes were blocked with 2% BSA, in PBS-T and incubated with 0.02 μg/mL of streptavidin-HRP (Jackson Immunoresearch) diluted in PBS-T, 0.2% BSA and detected with the Supersignal West pico chemiluminescent substrate (Pierce).

### Immunogenicity study

Proteins were heat treated at 80°C for 10 minutes or left untreated and immobilized on streptavidin-coated, 96-well plates (NUNC), at concentrations sufficient for complete saturation of the available binding surface/well (as determined by ELISA). Following three washes in PBS-T, plates were incubated with pooled sera from malaria-exposed Malawian adults or malaria-naïve UK individuals at a 1:1,000 dilution in PBS-T, 2% BSA followed by an alkaline phosphatase-conjugated anti-human IgG secondary antibody (Sigma) and detected as above.

## Results

### Identification of candidate *Plasmodium falciparum* merozoite cell surface and secreted proteins

With the aim of expanding an existing recombinant *P. falciparum* merozoite cell surface and secreted protein library, publicly available, genome-wide transcription microarray data of *P. falciparum* intra-erythrocytic stages were analysed [38,39]. To compile a list of merozoite extracellular proteins with possible roles in erythrocyte invasion, the transcription profiles of four well-established *P. falciparum* merozoite ligands (RH5, AMA1, EBA140, EBA175) that have all been previously implicated in erythrocyte invasion were examined [11,38-41]. It was observed that they all follow a similar expression pattern, passing through a minimum 20 ± 6 hours post invasion and peaking at approximately 42 ± 6 hours after invasion [38,39]. Of the 465 candidate blood stage genes that showed similar expression time windows, 207 encoded a predicted signal peptide and/or a single transmembrane domain/GPI anchor [28-30], suggesting they are likely cell surface or secreted proteins. Multi-pass membrane proteins were excluded from this list because these proteins are unlikely to be expressed in a soluble, secreted form. Within the 207 candidates, 31 were already represented in the existing merozoite library [27] and another 120 were excluded because of their predicted function (e g, involvement in lipid metabolism or nuclear localization) or protein domain content (e g, RNA or DNA binding motif) following gene ontology analysis using PlasmoDB [42], and protein domain mapping using Pfam [43]; also, 42 proteins were excluded due to their large size (>1,400 amino acids). In total, this bioinformatics analysis identified 14 putative secreted or membrane-tethered merozoite proteins. To this list, other members of the MSP3 [44,45], MSP7-like [46-48] and SERA paralogous protein families [49] were added, which were not present in the existing *P. falciparum* merozoite recombinant protein library [27]. Finally, the available literature was scanned to identify an additional seven merozoite cell surface and secreted proteins [50,51]. In total, 25 putative merozoite secreted or cell surface proteins were chosen for recombinant expression, two of which contained putative GPI anchors, and 23 contained no predicted membrane anchor (Table 1).

**Table 1:** The putative merozoite cell surface and secreted proteins that were chosen for recombinant expression

**No**	**Name**	**Accession number**	**Type**	**Region targeted for recombinant expression**	**Exp. levels**
1**¥**	rhoptry-associated membrane antigen (RAMA)	MAL7P1.208	GPI	Y17–S840	Medium
2	Prohibitin, putative	PF08_0006	Secreted	L20–F272	Low
3#	Conserved *Plasmodium* protein, unknown function	PF10_0166	Secreted	Y25–E310	High
4	GLURP	PF10_0344	Secreted	K24–I1233	Low
5	MSP3.5	PF10_0350	Secreted	A20–F710	High
6*	MSP3.6	PF10_0351	Secreted	N22–P566	Medium
7*	MSRP5	PF13_0191	Secreted	N22–I459	Medium
8*	MSRP4	MAL13P1.173	Secreted	D22–Q309	High
9**¥**	MSP8	PFE0120c	GPI	E26–S576	Low
10#	Conserved *Plasmodium* protein, unknown function	PF13_0125	Secreted	N20–S292	High
11	Conserved *Plasmodium* protein, unknown function	PF14_0044	Secreted	Q21–K290	N/D
12	Merozoite-associated tryptophan-rich antigen, putative	PFA0135w	Secreted	I25–K276	High
13	LCCL domain-containing protein	PFA0445w	Secreted	K22–I1029	N/D
14	SERA1	PFB0360c	Secreted	M1–V997	N/D
15**¥**	SERA2	PFB0355c	Secreted	E23–V1105	Medium
16	SERA3	PFB0350c	Secreted	T23–I930	Medium
17	SERA4	PFB0345c	Secreted	S26–V962	High
18	SERA5	PFB0340c	Secreted	T23–V997	High
19	SERA6	PFB0335c	Secreted	N25–V1031	Low
20**¥**	SERA7	PFB0330c	Secreted	Q23–V946	Low
21*	SERA9	PFI0135c	Secreted	E23–V932	Medium
22**¥**	Conserved *Plasmodium* protein, unknown function	PFA0210c	Secreted	Y24–D466	Low
23	Conserved *Plasmodium* protein, unknown function	PFB0475c	Secreted	L23–D446	Low
24	Cysteine-rich protective antigen (CyRPA)	PFD1130w	Secreted	D29–E362	High
25	RIPR	PFC1045c	Secreted	I20-N1086	Low

Protein sequences from 3D7 reference *P. falciparum* strain were truncated to remove endogenous signal peptides and sequences corresponding to GPI anchors. The region of each protein that was targeted for recombinant expression is shown. Expression levels are given as a guide only because of the significant batch-to-batch variability of transient transfection, and grouped into "high" (between 30 μM and 100 μM after purification), "medium" (1 μM and 30 μM) and "low" (0.01 μM and 1 μM). No detectable expression (N/D) was obtained for PFA0445w, PF14_0044 and SERA1.

**¥**Proteins selected from [51].

*Proteins included to complete the set of proteins belonging to the MSP3, MSP7-like and SERA families.

**#**Proteins chosen from [50]. All other proteins were short-listed from bioinformatic analysis of transcription microarray data [38,39]. GPI, GPI-anchored.

To design the expression constructs, the entire predicted extracellular domain was selected between the signal sequence and the GPI-anchor, if present. Any predicted N-linked glycosylation sites were systematically removed by substituting alanine for serine/threonine at these sites. Expression constructs were made by gene synthesis and codon optimized for expression in human cells. All expression plasmids are publicly available through Addgene, a not-for-profit, open access plasmid repository [36].

### Expression and purification of an expanded *Plasmodium falciparum* merozoite protein library

All proteins were expressed in HEK293E cells as soluble fusion proteins that contained a C-terminal rat Cd4(d3 + 4)-hexahistidine tag for purification and could be optionally monobiotinylated by cotransfecting a secreted version of the *E. coli* BirA enzyme [33]. To purify many proteins in parallel for comparative screening, a custom-built protein purification system was employed, that enables the simultaneous purification of up to 96 His-tagged proteins, even from large (>50 mL) tissue culture volumes [33].

Following purification, recombinant proteins were quantitated by ELISA and while expression levels varied significantly between individual proteins, most were purified to micromolar levels (Table 1). Overall, detectable expression was obtained for 22 out of 25 (88%) proteins. Both GLURP and SERA6, although detected by ELISA, were expressed at levels that were too low to include in further analysis and three proteins (PF14_0044, PFA0445w, SERA1) remained undetectable by ELISA despite repeated transfections.

**Figure 1:**
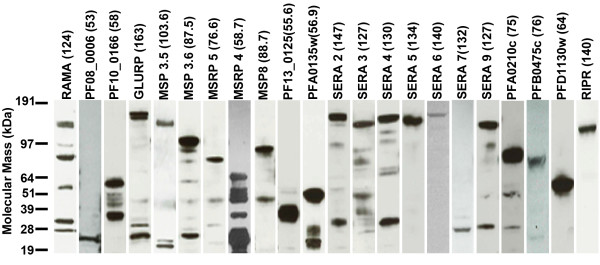
**The majority of recombinant merozoite extracellular proteins are expressed at their expected size.** One microgram of each purified biotinylated merozoite protein (as estimated by the absorbance at 280 nm) was resolved under reducing conditions by SDS-PAGE, blotted, and probed using streptavidin-HRP. The expected molecular mass of each recombinant protein is indicated in brackets above each lane, including the Cd4-6xHis tag (25 kDa).

To assess their integrity, the recombinant proteins were resolved by SDS-PAGE, and the presence of the C-terminal biotin tag detected by Western blotting (Figure 1). A band consistent with the full-length ectodomain was obtained for 20 out of 25 proteins (80%); in some cases, smaller bands were also evident, most likely due to proteolytic processing. For SERA7 and PF08_0006, bands of ~30 and ~25 kDa were detected, respectively, suggesting complete cleavage at the C-terminus of the protein. Collectively, these observations establish the successful expression of 18/25 (72%) recombinant *P. falciparum* merozoite proteins at a size consistent with a full-length recombinant protein at usable amounts.

### Members of the recombinant merozoite protein library proteins are immunoreactive and carry heat-labile epitopes

**Figure 2:**
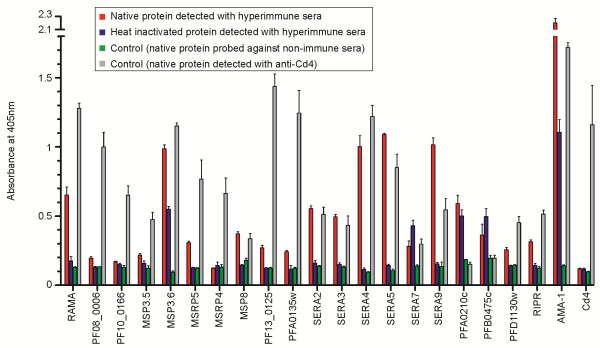
**The merozoite recombinant proteins are immunoreactive against hyperimmune sera.** The immunoreactivity of the recombinant *P. falciparum* merozoite proteins was tested using pooled sera from malaria-exposed Malawian adults (red bar) or malaria-naïve adults (green bar). The reduced immunoreactivity of immune sera to heat-denatured antigens (blue bar) demonstrates the presence of heat-labile (conformational) epitopes. All proteins except one (MSRP4) were identified as being immunoreactive as assessed by immunoreactivity >3 SD above negative control (green bar). AMA-1 and Cd4 were used as the positive and negative control, respectively. Data points are shown as mean ± s.d.; *n* = 3.

In nature, protective antibodies largely recognize proteins in their native conformation; therefore, to examine whether the recombinant merozoite library proteins were correctly folded, their immunoreactivity against hyperimmune sera from adults living in malaria-endemic regions was tested. All 20 proteins from the library expressed at useable levels were arrayed on a streptavidin-coated, microtitre plate and their relative immunoreactivity to pooled sera from Malawian adults was compared to that from malaria-naïve individuals [52]. All but one (MSRP4) of the proteins were immunoreactive (Figure 2). Strikingly, strong immunoreactivity was observed for most of the SERA proteins, consistent with previous observations [49].

To demonstrate that serum antibodies were recognizing conformational epitopes within the protein library, all recombinant proteins were denatured by heat treatment before being captured via their biotin tag. For 16 of the 20 proteins (all but MSRP4, SERA7, PFA0210c, and PFB0475c) the immunoreactivity decreased significantly when the proteins were heat inactivated, establishing that the antigens contain heat-labile epitopes. These data show that the merozoite recombinant proteins are correctly folded and at least in part mimic the native protein conformation.

## Discussion

The technical difficulties in expressing *Plasmodium* proteins in a recombinant functional form has presented difficulties both for basic malaria research and vaccine development [12]. Here, an approach based on a mammalian expression system has been utilized to significantly expand a recombinant library consisting of recombinant *P. falciparum* merozoite extracellular proteins from 42 to 62 members [27]. A high-throughput, custom-made purification platform was successfully used to purify recombinant proteins from large volumes of tissue culture supernatants to permit systematic comparative studies of purified antigens [33].

While the overall success rate of expression was high, consistent with previous experience of the HEK293 system [27,53-57], a few merozoite proteins still failed to express, or were expressed at low levels. Although the genes were codon-optimized for human cells, *P. falciparum* proteins are unusual because they are enriched in asparagine, glutamic acid and lysine, and often contain homopolymeric stretches of amino acids. Indeed, it has been recently demonstrated that the stability of several *Plasmodium* proteins depends upon their association with heat shock proteins which act as molecular chaperones [58]. Therefore, recombinant protein expression in the HEK293 system could be further enhanced by the presence of *Pf*Hsp110c, which has been proposed to be a protein-stabilizing chaperone [58].

Using sera from malaria-immune adults, it has been shown that the expressed recombinant proteins contained heat-labile epitopes suggesting that they adopt their native conformation and are likely to be biochemically active. A number of proteins (e g, PF10_0166, PFA0135w) showed little serological response in comparison to the negative control. While these proteins may not contain epitopes present in the native protein, the possibility that the native proteins are poorly immunogenic and do not induce a strong antibody response *in vivo*, cannot be excluded. For example, previous studies reported that only 23% of immune sera examined contained specific serum IgG antibodies against PFA0135w, suggesting that this protein does not normally elicit strong humoral responses [59]. Interestingly, RIPR, which binds to *Pf*RH5 [60], is also among the group of generally low responders. *Pf*RH5 is a high priority vaccine target as it plays an essential and universal role in erythrocyte invasion [53,57,61-63], yet it is poorly immunogenic *in vivo*[61], possibly due to its late release onto the merozoite surface during erythrocyte invasion. RIPR may be similarly masked from the host immune system, but further work with this antigen is clearly required.

## Conclusion

A library of recombinant *P. falciparum* proteins has been expanded and characterized with the eventual aim of compiling a set that is representative of the merozoite surface. These plasmids, which are freely available to the global research community through Addgene [36], will be a valuable resource for basic research and aid the efforts to develop an effective malaria vaccine.

## Competing interests

The authors declare that they have no competing interests.

## Authors’ contributions

ZAZ, JCR and GJW conceived the study and ZAZ performed the experiments. ZAZ wrote the paper with contributions from JCR and GJW. All authors read and approved the final manuscript.
